# Evaluating Brain Activity in Patients With Chronic Disorders of Consciousness After Traumatic Brain Injury Using EEG Microstate Analysis During Hyperbaric Oxygen Therapy

**DOI:** 10.1111/cns.70220

**Published:** 2025-01-20

**Authors:** Long Xu, Jiameng Wang, Cong Wang, Qianqian Ge, Ziqi Ren, Chen He, Yun Liu, Bo Wang, Yaling Liu, Lianbi Xue, Jianghong He, Xudong Zhao, Qiuhong Yu

**Affiliations:** ^1^ Department of Neurosurgery, Beijing Tiantan Hospital Capital Medical University Beijing China; ^2^ China National Clinical Research Center for Neurological Diseases Beijing China; ^3^ Institute of Artificial Intelligence Hefei Comprehensive National Science Center Hefei China; ^4^ Department of Hyperbaric Oxygenation, Beijing Tiantan Hospital Capital Medical University Beijing China; ^5^ State Key Laboratory of Brain and Cognitive Science Institute of Biophysics, Chinese Academy of Sciences Beijing China; ^6^ CAS Center for Excellence in Brain Science and Intelligence Technology Beijing China; ^7^ University of Chinese Academy of Sciences Beijing China

**Keywords:** EEG, hyperbaric oxygen, microstate, prolonged DOCs, traumatic brain injury

## Abstract

**Background:**

Hyperbaric oxygen (HBO) therapy is an efficacious intervention for patients with prolonged disorders of consciousness (pDOC). Electroencephalographic (EEG) microstate analysis can provide an assessment of the global state of the brain. Currently, the misdiagnosis rate of consciousness‐level assessments in patients with pDOC is high. Therefore, we aimed to assess the consciousness levels and outcomes of patients by analyzing changes in EEG signals during HBO therapy.

**Methods:**

EEG data were collected from 32 patients with traumatic brain injury before and after 20 min of HBO therapy. EEG data were obtained during HBO therapy sessions. Modified k‐means clustering was used to segment EEG signals into microstates. A paired sample t test was used to compare the microstate characteristics before and during HBO therapy.

**Results:**

The duration, occurrence, and coverage of microstate D significantly increased in the minimally conscious state (MCS) group after therapy. Significant increases in the same parameters were observed in microstate A among patients in the unresponsive wakefulness state group. Furthermore, patients with greater improvements in Coma Recovery Scale‐Revised scores (i.e., improvements of more than three points) showed significant increases in the duration, occurrence, and coverage of microstate D. Both the MCS group and the improvement group presented significant increases in the duration, occurrence, and coverage of microstate D during therapy.

**Conclusions:**

Microstate D may be associated with the recovery of consciousness levels in patients. This study verified the safety and feasibility of real‐time EEG during HBO therapy for patients with pDOC. The changes in EEG microstate characteristics during HBO therapy can serve as a significant complement to electroencephalographic assessment indices for patients with pDOC and may be useful for predicting the recovery of consciousness levels.

## Graphical abstract

During HBO therapy, patients with pDOC exhibit regular changes in EEG microstates, which could serve as indicators to assess consciousness levels, and the characteristics of microstate D may hold predictive value for the potential recovery of consciousness.

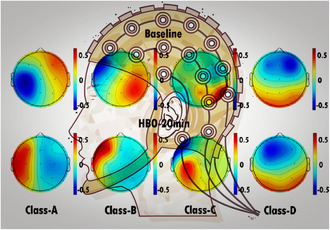

## 1 Introduction

Globally, traumatic brain injury (TBI) is the leading cause of neurological conditions; furthermore, TBI is a substantial contributor to disability and mortality rates and thus imposes a substantial burden on healthcare systems worldwide [1]. Additionally, TBI is a common cause of prolonged disorders of consciousness (pDOC), a condition characterized by the absence of functional communication or purposeful movement for more than 4 weeks following injury, as defined by clinical and behavioral assessments, including tools such as the Coma Recovery Scale‐Revised (CRS‐R). pDOC typically arises from a combination of direct and indirect forces, including but not limited to falls, traffic accidents, blunt force trauma, and other impact‐related incidents [2].

In many countries, accurately assessing a patient's level of consciousness and prognosis holds significant importance for guiding treatment decisions and allocating medical resources. The use of subjective scales based solely on verbal responses to assess consciousness levels and predict prognosis often results in high misdiagnosis rates [3, 4]. Recent studies have shown that behavioral assessments, such as the CRS‐R, are prone to misdiagnosis, with error rates reaching up to 36% in patients with DOC due to fluctuations in responsiveness and limited sensitivity to covert awareness [5]. Therefore, there is an ongoing transition toward a novel diagnostic and prognostic assessment staging that integrates behavioral scales with neuroimaging and neurophysiology. As a neuroimaging modality, electroencephalography (EEG) stands out for its cost‐effectiveness and ease of operation [6]. EEG measures the electric field strength on the scalp and thus mirrors the functioning of different brain regions [7]. Presently, a range of EEG‐derived metrics serve as critical biomarkers in the diagnosis and management of various conditions [8]. When patients transition from the unresponsive wakefulness state (UWS, previously referred to as the vegetative state [VS]) to the minimally conscious state (MCS), there is a turning point in the EEG features [9]. Research suggests that specific EEG patterns serve as independent predictors of improved consciousness upon discharge among patients in the UWS [10]. Combining EEG with behavioral assessments offers a more objective and standardized approach to diagnosing disorders of consciousness, potentially reducing variability, and improving diagnostic accuracy.

Within the domain of EEG analysis, microstate analysis serves as a powerful tool for feature extraction and subsequent examinations. Microstates are brief intervals (lasting between 80 and 120 ms) during which the electric potential of the scalp exhibits relative stability, followed by a transition [11]. Microstate analysis is distinctive in terms of its comprehensive consideration of signals from all electrodes, and it can be used to capture a wealth of syntactic and sequential data [12]. EEG microstates have been identified as promising candidates for endophenotyping or biomarker analysis, thus offering insights into the relationships among anesthesia, sleep, and microstates [13, 14, 15].

Previous studies have commonly categorized microstates into four distinct types, each linked to specific brain regions and networks. Microstate A is associated primarily with the bilateral superior and middle temporal gyri, regions integral to the auditory system. Studies suggest that this microstate is closely connected to the auditory network, reflecting processes involved in auditory information input and analysis [16]. Microstate B is related to brain areas responsible for visual processing and is thought to correspond to the visual network. Alterations in microstate B are often among the earliest indicators of damage or changes within the human visual system [17]. Microstate C involves regions such as the posterior anterior‐cingulate cortex, bilateral inferior frontal gyrus, and right anterior insula. It is associated with the salience network and plays a crucial role in facilitating transitions between the central executive network and the default mode network [17]. Microstate D corresponds to areas within the right dorsal and ventral frontoparietal cortex. It is linked to the central executive network, which governs advanced cognitive functions such as decision‐making and problem‐solving [17].

Many studies have demonstrated the positive effects of hyperbaric oxygen (HBO) therapy in patients with pDOC [12, 18, 19]; however, the detection of brain function during HBO therapy has rarely been reported. Our preliminary research confirmed the feasibility of real‐time EEG monitoring during HBO therapy in a study of 41 patients with varying degrees of pDOC caused by factors such as TBI, anoxic events, and stroke [12]. Our preliminary study indicated that HBO therapy has a specific activating effect on attention and cognitive control in patients and causes increased activity in the primary sensory cortex (temporal lobe and occipital lobe). However, it is unclear whether EEG changes during HBO therapy are correlated with patient outcomes. Therefore, the main purpose of this study was to investigate the correlation between EEG microstate changes and outcomes in TBI patients receiving HBO therapy. We aimed to explore the feasibility of real‐time EEG detection during HBO therapy and its role in assessing brain function. Changes in EEG microstates during HBO therapy may serve as complementary electrophysiological indicators for grading consciousness levels and in prognostic assessments.

## 2 Materials and Methods

**FIGURE 1:**
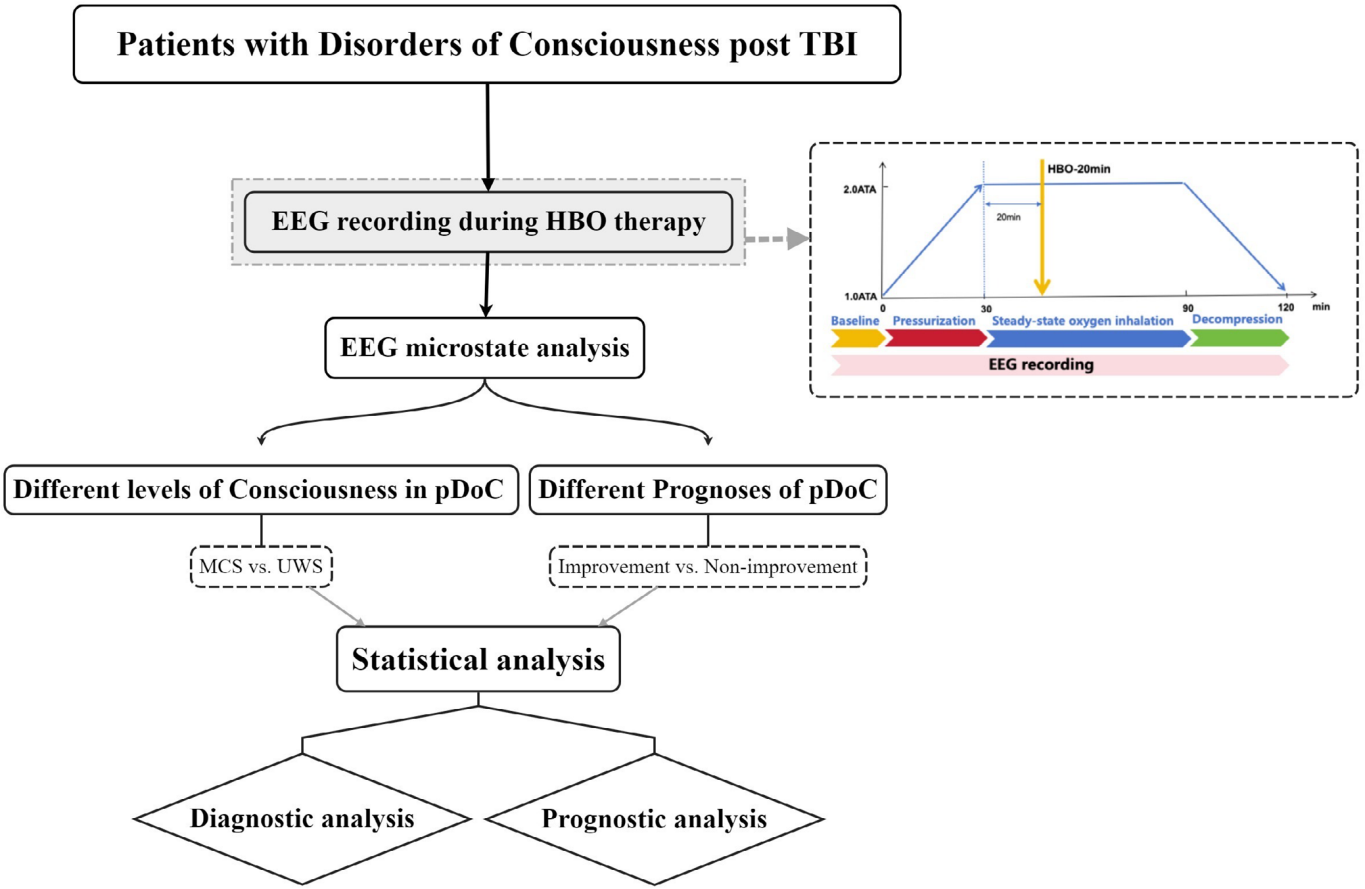
Study design of EEG microstate analysis in consciousness disorders among 32 patients with TBI. Thirty‐two TBI patients with prolonged disorders of consciousness (pDOC) were included. EEG data were recorded before and during HBO therapy and analyzed using microstate analysis. Patients were grouped based on their consciousness level (MCS vs. UWS) and prognosis (improvement vs. nonimprovement) to explore diagnostic and prognostic implications of EEG microstate changes.

This study included 32 patients with TBI from November 2020 to January 2022 at Beijing Tiantan Hospital, Capital Medical University. The patients were grouped on the basis of their level of consciousness upon admission and their recovery of consciousness during follow‐up. To further investigate the relationship between the level of consciousness and prognosis, comparisons were made between patients in the MCS group and those in the improvement group (Figure 1).

The inclusion criteria for all enrolled patients were as follows: (1) a confirmed diagnosis of DOC, (2) age between 18 and 80 years, (3) an onset time exceeding 1 month, (4) a stable consciousness level, with no improvement or deterioration for at least 4 weeks prior to enrolment, and (5) consent from family members to undergo HBO therapy, with a signed informed consent form. The exclusion criteria were as follows: (1) inability to tolerate HBO therapy, pDOC caused by neurodegenerative diseases (e.g., Alzheimer's disease, Lewy body dementia) and malignant brain tumor surgery, (2) coma caused by exacerbation of systemic diseases or expected survival time, (3) duration of illness < 1 month, (4) epileptic seizures that were difficult to control, (5) treatment with experimental drugs or equipment, and (6) untreated tension pneumothorax or other conditions that the HBO physician deemed inappropriate for treatment.

Follow‐up assessments were conducted using the Coma Recovery Scale‐Revised (CRS‐R) to gauge patient outcomes at 6 months after treatment.

The study was conducted in accordance with the Declaration of Helsinki of the World Medical Association and approved by the Ethics Committee of Beijing Tiantan Hospital (No. KY2023–175‐03). Before inclusion, the researcher fully informed each patient's legal guardian of the study protocol and obtained informed consent from them.

### 2.1 Clinical Evaluation

The patient's level of consciousness was assessed using the CRS‐R [20], a standardized tool that evaluates six subscales: auditory, visual, motor, oromotor/verbal, communication, and arousal functions. Two trained clinicians independently administered the CRS‐R. The CRS‐R was administered at least three times 1 week before HBO therapy to determine the patient's baseline level of consciousness.

### 2.2 HBO Therapy Procedure

The HBO therapy described in this investigation took place within the specialized intensive care unit of the Hyperbaric Oxygen Department at Beijing Tiantan Hospital, affiliated with Capital Medical University. The protocol stipulated the application of hyperbaric conditions at 0.2 MPa—a significant increase from standard atmospheric pressure (0.1 MPa). Each therapy session included a total duration of 120 min, consisting of a 30‐min pressurization phase, a 60‐min steady‐state oxygen inhalation phase, and a 30‐min decompression phase. The facility was outfitted with critical medical apparatuses, including a ventilator and a sputum suction unit, to facilitate comprehensive care. Moreover, continuous ECG surveillance was implemented, supported by the presence of medical personnel throughout the therapy, to ensure optimal patient safety. Additionally, the oxygen and carbon dioxide levels within the chamber were vigilantly monitored to ensure that they remained within established normative limits.

### 2.3 EEG Acquisition and Processing

EEG data were acquired through a Nicolet EEG apparatus, which utilizes 19 channels for recording purposes. This process was conducted in tandem with HBO therapy sessions, employing a sampling frequency of 500 Hz to ensure precise data capture. To maintain the integrity of the recordings, electrode impedance was kept below 5 kΩ throughout the data collection phase. Additionally, electrodes A1 and A2, located at the bilateral mastoids, were utilized as reference points during the acquisition process. EEGs were recorded continuously throughout the therapy, and we analyzed baseline data obtained before pressurization and data recorded 20 min after the steady‐state oxygen phase.

Data preprocessing was executed via plugins from the EEGLAB toolbox within the MATLAB environment. The initial phase involved the application of a 50‐Hz notch filter, in conjunction with a 1‐ to 45‐Hz bandpass filter, tailored to the specific conditions under which the data were acquired. Next, the superfluous electrodes, notably the bilateral mastoid electrodes designated A1 and A2, were removed from the dataset. The processed data were then segmented into epochs of 3 s in duration. The subsequent phase focused on artifact mitigation within the signal. An initial manual inspection was conducted to identify and remove the majority of artifacts, after which an independent component analysis (ICA) algorithm was deployed for further refinement. This methodology was strategically chosen to minimize the impact on the intrinsic characteristics of the EEG signals while effectively eliminating artifacts associated with electrooculographic, electromyographic, and ECG activities.

### 2.4 EEG Microstate Analysis

To investigate the microstates within the preprocessed EEG data, we applied an analytic framework that aligns with established research protocols. Our initial step involved the computation of global field power (GFP), which is a measurement of the brain's electric field strength at a given instant. This parameter is pivotal in gauging the brain's response to external stimuli and in delineating fluctuations in neural activity [21].

For the microstate analysis, we utilized the peak GFP values to define discrete EEG states, which were represented through topographic maps. These maps were subjected to cluster analysis to segregate them into distinct categories using the modified k‐means algorithm from the MATLAB toolbox [22]. The algorithm partitions the EEG data into a predefined number of clusters and iteratively adjusts the sample allocation to enhance cluster validity [23].

This clustering process identified four distinct microstates (labeled A, B, C, and D), and the characteristics of these microstates were primarily discerned through their respective topographic configurations [16, 24]. After clustering, we computed several key parameters for each microstate across the patient cohort, including the mean microstate duration (MMD), the ratio of total time covered (RTT), and the global explained variance (GEV), along with metrics such as transition probabilities, mean occurrence rates, and average GFP values for each microstate. These calculations offer a comprehensive quantification of the microstate attributes and their temporal dynamics within the EEG data.

### 2.5 Statistical Analysis

Statistical analyses were performed using SPSS 25 software. Our statistical methods are similar to those used in other microstate studies [11, 14, 16, 21, 25, 26]. The normality of the data was evaluated through the Shapiro–Wilk test. Data that did not follow a normal distribution were analyzed using the Wilcoxon signed‐rank test. Differences in microstate characteristics before and during HBO therapy were examined via paired sample t tests. The threshold for statistical significance was *p* < 0.05.

## 3 Results

### 3.1 Patients

A total of 32 patients with TBI were recruited at Beijing Tiantan Hospital, Capital Medical University, from November 2020 to January 2022. The ages of the patients ranged from 21 to 76 years (49.8 ± 15.4), and 22 male patients (49.2 ± 16.8 years) and 10 female patients (51.2 ± 12.4 years) were included. The time since injury ranged from 1 to 24 months (6.0 ± 5.4), the CRS‐R scores before HBO therapy ranged from 2 to 15 (6.9 ± 3.3), and the CRS‐R scores at 6 months after HBO therapy ranged from 3 to 23 (10.4 ± 6.5) (Table 1).

**TABLE 1:** Details of the 32 patients who participated in real‐time EEG monitoring during HBO therapy.

Patient	Sex	Age (years)	Consciousness level	Emergency surgery	Time since injury (months)	CRS‐R (admit)	CRS‐R (follow‐up)	ΔCRS‐R
1	M	76	VS/UWS	Yes	1.0	5 (011102)	13 (234112)	8
2	M	64	VS/UWS	Yes	5.0	4 (101101)	8 (311102)	4
3	M	22	MCS	Yes	1.0	8 (113102)	23 (456323)	15
4	F	58	MCS	No	3.0	9 (132102)	9 (132102)	0
5	F	31	MCS	Yes	8.0	10 (133102)	20 (456122)	10
6	M	65	VS/UWS	No	7.0	4 (010102)	6 (111102)	2
7	F	62	VS/UWS	Yes	4.0	6 (111102)	16 (234313)	10
8	F	57	VS/UWS	Yes	4.0	6 (012102)	7 (112102)	1
9	M	63	MCS	Yes	12.0	9 (033102)	10 (133102)	1
10	M	35	MCS	No	2.0	8 (113102)	23 (456323)	15
11	M	50	VS/UWS	No	3.0	7 (112102)	7 (112102)	0
12	F	39	MCS	Yes	1.0	15 (345102)	23 (456323)	8
13	F	65	VS/UWS	Yes	2.0	5 (112100)	5 (112100)	0
14	M	43	VS/UWS	Yes	4.0	5 (002102)	5 (002102)	0
15	M	46	VS/UWS	No	5.0	5 (011102)	23 (456323)	18
16	F	34	MCS	Yes	2.0	14 (344102)	16 (345103)	2
17	M	47	MCS	Yes	12.0	15 (452202)	15 (452202)	0
18	M	36	VS/UWS	No	24.0	7 (112102)	8 (212102)	1
19	M	33	VS/UWS	Yes	4.0	7 (112102)	7 (112102)	0
20	M	53	MCS	Yes	2.0	11 (233102)	11 (233102)	0
21	M	64	VS/UWS	Yes	12.0	7 (121102)	9 (132102)	2
22	M	56	MCS	Yes	6.0	10 (133102)	10 (133102)	0
23	F	56	VS/UWS	Yes	12.0	4 (002002)	4 (002002)	0
24	M	21	VS/UWS	Yes	3.0	4 (001102)	5 (102002)	1
25	M	68	VS/UWS	No	5.0	6 (102102)	6 (102102)	0
26	F	62	VS/UWS	Yes	11.0	3 (002100)	3 (002100)	0
27	M	32	VS/UWS	No	8.0	3 (000102)	5 (101102)	2
28	M	21	VS/UWS	Yes	4.0	6 (111102)	17 (453122)	11
29	M	67	VS/UWS	Yes	2.0	5 (011102)	7 (112102)	2
30	F	48	VS/UWS	No	19.0	4 (001102)	4 (001102)	0
31	M	56	VS/UWS	No	1.5	6 (102102)	6 (102102)	0
32	M	64	VS/UWS	Yes	3.0	2 (001100)	3 (002100)	1

*Note:* Thirty‐two patients were included in the study; they were aged 21–76 years (49.8 ± 15.4), the time since injury ranged from 1 to 24 months (6.0 ± 5.4), the pretreatment CRS‐R scores (admit) ranged from 2 to 15 (6.9 ± 3.3), and the posttreatment CRS‐R scores (follow‐up) ranged from 3 to 23 (10.4 ± 6.5). ΔCRS‐R = CRS‐R (follow‐up)—CRS‐R (admit). Twenty‐two male patients (age: 49.2 ± 16.8) and 10 female patients (age: 51.2 ± 12.4) were included in the study.

Abbreviations: CRS‐R, Coma Recovery Scale‐Revised; F, female; M, male; MCS, minimally conscious state; VS/UWS, vegetative state/unresponsive wakefulness syndrome.

### 3.2 EEG Microstate Changes in Patients With TBI During HBO Therapy

A total of 32 individuals suffering from TBI were systematically enrolled in this study. These participants completed an intervention involving a single session of HBO therapy that was simultaneously monitored using EEG. Notably, the intervention proceeded without the occurrence of any adverse effects, such as barotrauma affecting the middle ear or lungs. The HBO therapy session was conducted within the hyperbaric chamber, and the treatment was administered smoothly, with no recorded instances of abrupt changes in respiratory function or blood pressure; no reports of suffocation triggered by excessive phlegm; and no incidents of seizures, fever, or other undesirable events.

**FIGURE 2:**
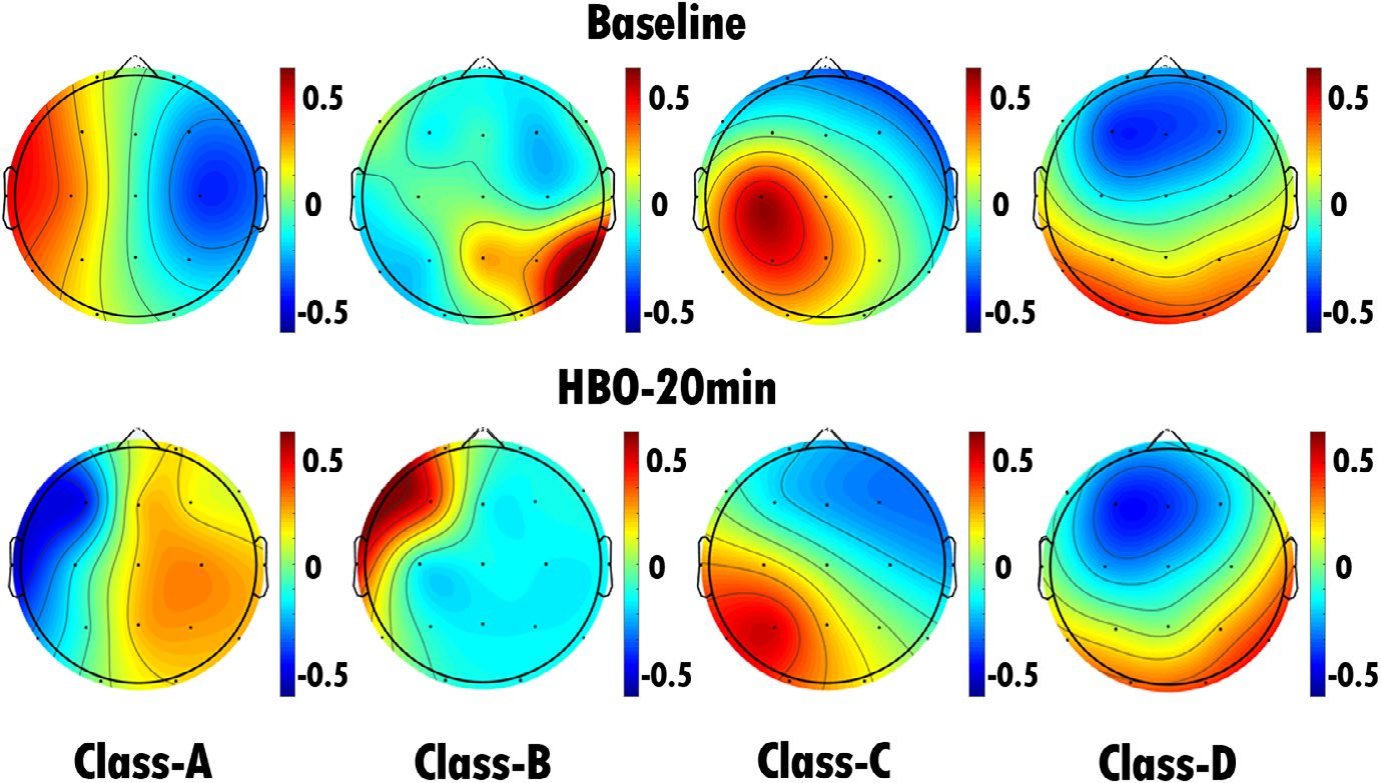
EEG microstate topography of all patients with TBI (32 patients). The top row shows the EEG microstate topography map of 32 patients with TBI before HBO therapy, and the bottom row shows the EEG microstate topography map of 32 TBI patients after 20 min of HBO therapy. There was a pronounced change in the topographical map of microstate C before and during HBO therapy, with varying degrees of changes in the topographical maps of the other microstates.

We conducted EEG microstate analysis before and during HBO therapy. Following the approach outlined by Britz, Van De Ville, and Michel and others [27], we applied a microstate clustering algorithm to fit the pre‐ and posttreatment EEG microstates, ultimately defining them as microstates A, B, C, and D (Figure 2). The microstate class orientations were specified as follows: (A) right‐frontal left‐posterior, (B) left‐frontal right‐posterior, (C) anterior–posterior, and (D) frontocentral extreme. Polarity was disregarded in the microstate analysis. By comparing the topographical maps of the microstates, we observed that there was a pronounced change in the topographical map of microstate C before and during HBO therapy, with varying degrees of changes in the topographical maps of the other microstates.

Prior studies have demonstrated that neuromodulation therapies can modulate EEG microstate dynamics. For example, TMS has been shown to induce significant changes in EEG microstates, particularly in microstates C and D, reflecting alterations in large‐scale brain networks that are associated with improvements in cognitive and emotional function, as observed in patients with subacute stroke [25]. Similarly, tDCS studies have demonstrated its ability to modulate EEG microstate parameters, particularly by altering the duration and transitions of microstates, which may improve network efficiency and functional connectivity, as well as alleviate symptoms in individuals with neurological and psychiatric conditions such as obsessive‐compulsive disorder [28]. Compared to these approaches, HBO therapy, as evaluated through EEG microstate analysis, offers a distinct mechanism of action. While TMS and tDCS target cortical excitability or connectivity, HBO therapy appears to induce broader effects by enhancing oxygen delivery and stimulating neurophysiological processes. The pronounced changes in microstate C observed in our study suggest that HBO therapy may complement other neuromodulation therapies in modulating neural activity in patients with pDOC.

We also conducted specific analyses on different enrolled patients. The process of collecting EEG data from patients receiving HBO therapy is referred to as HBO‐EEG.

### 3.3 HBO‐EEG Microstates Between Different Levels of Consciousness

Table 1 presents demographic and clinical information, including sex, age, etiology, and CRS‐R scores, for the included patients. Patients were categorized into two groups on the basis of their level of consciousness at admission: the UWS group (22 patients) and the MCS group (10 patients). We conducted EEG microstate analysis on patients in both the UWS and MCS groups before and during HBO therapy, thus generating microstate topographies at baseline and 20 min after HBO therapy.

**FIGURE 3:**
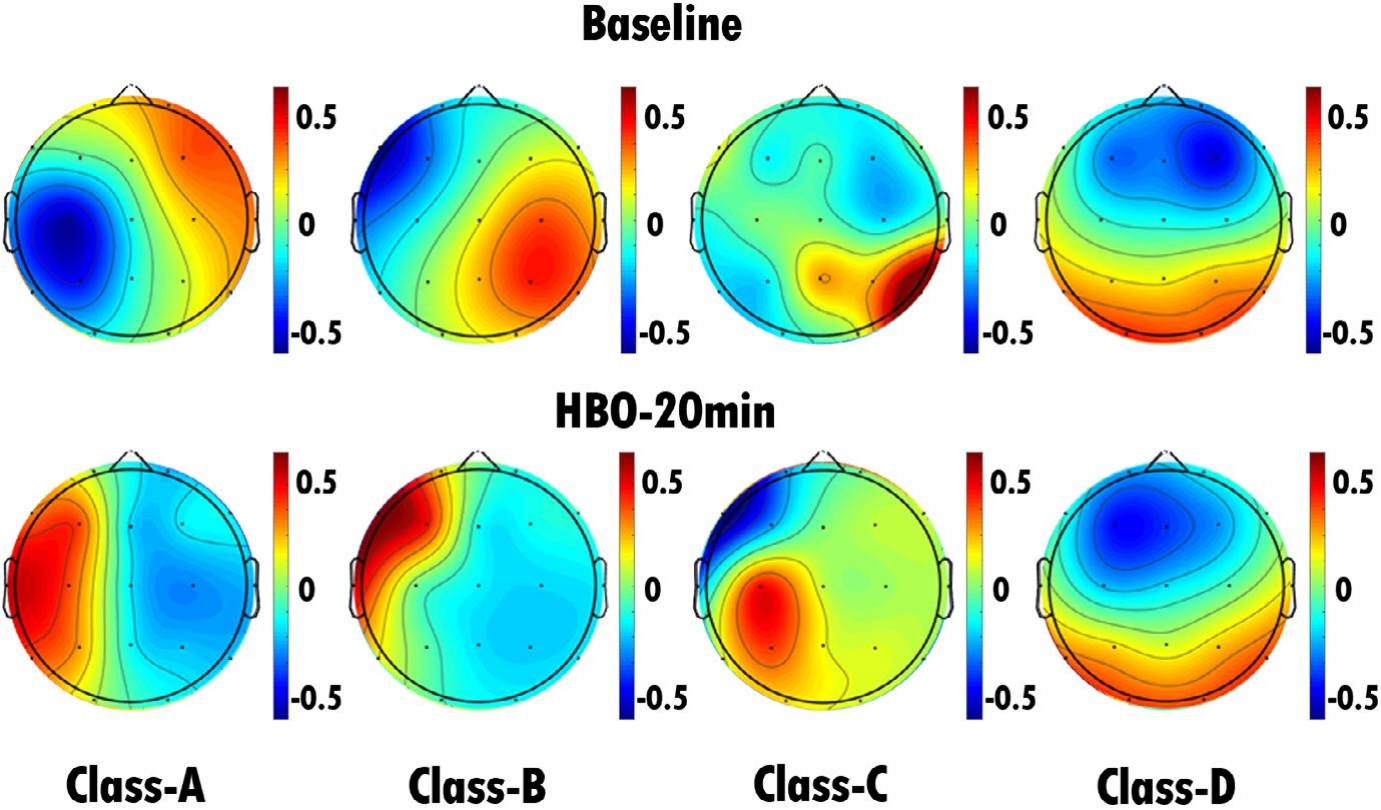
EEG microstate topography of patients in the MCS group (10 patients). The top row shows the EEG microstate topography map of the patients before HBO therapy, and the bottom row shows the EEG microstate topography map of the patients after 20 min of HBO therapy. We found that the duration, occurrence, and contribution of microstate D were significantly greater (*p* < 0.05) after 20 min of HBO therapy than before HBO therapy. There was a significant decrease in the coverage of microstate A, whereas there were significant increases in the duration, occurrence, and coverage of microstate D.

**FIGURE 4:**
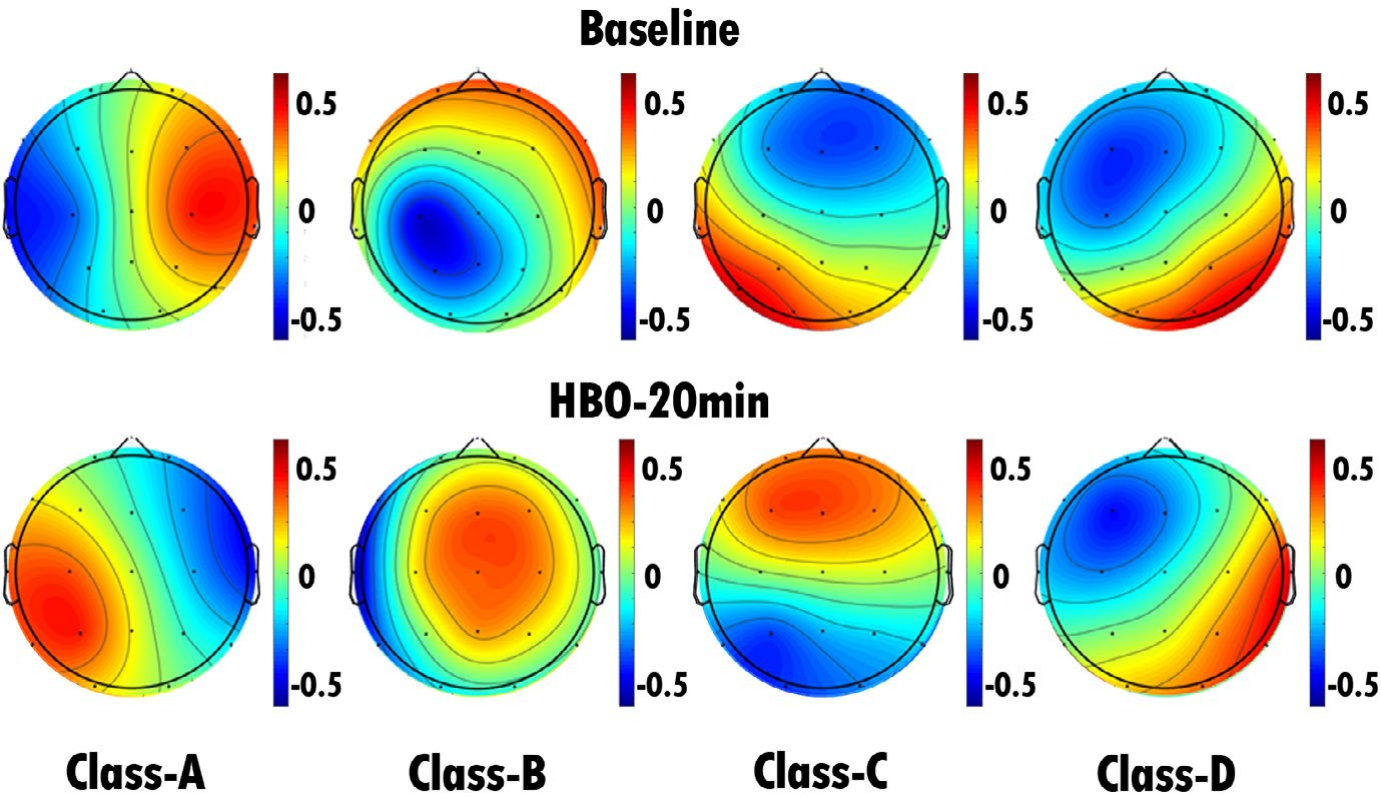
EEG microstate topography of patients in the UWS group (22 patients). The top row shows the EEG microstate topography map of the patients before HBO therapy, and the bottom row shows the EEG microstate topography map of the patients after 20 min of HBO therapy. We found that the duration, occurrence, and contribution of microstate A significantly increased (*p* < 0.05) after 20 min of HBO therapy compared with the pretreatment values. There was a significant increase in the duration, occurrence, and coverage of microstate A after 20 min of treatment.

**FIGURE 5:**
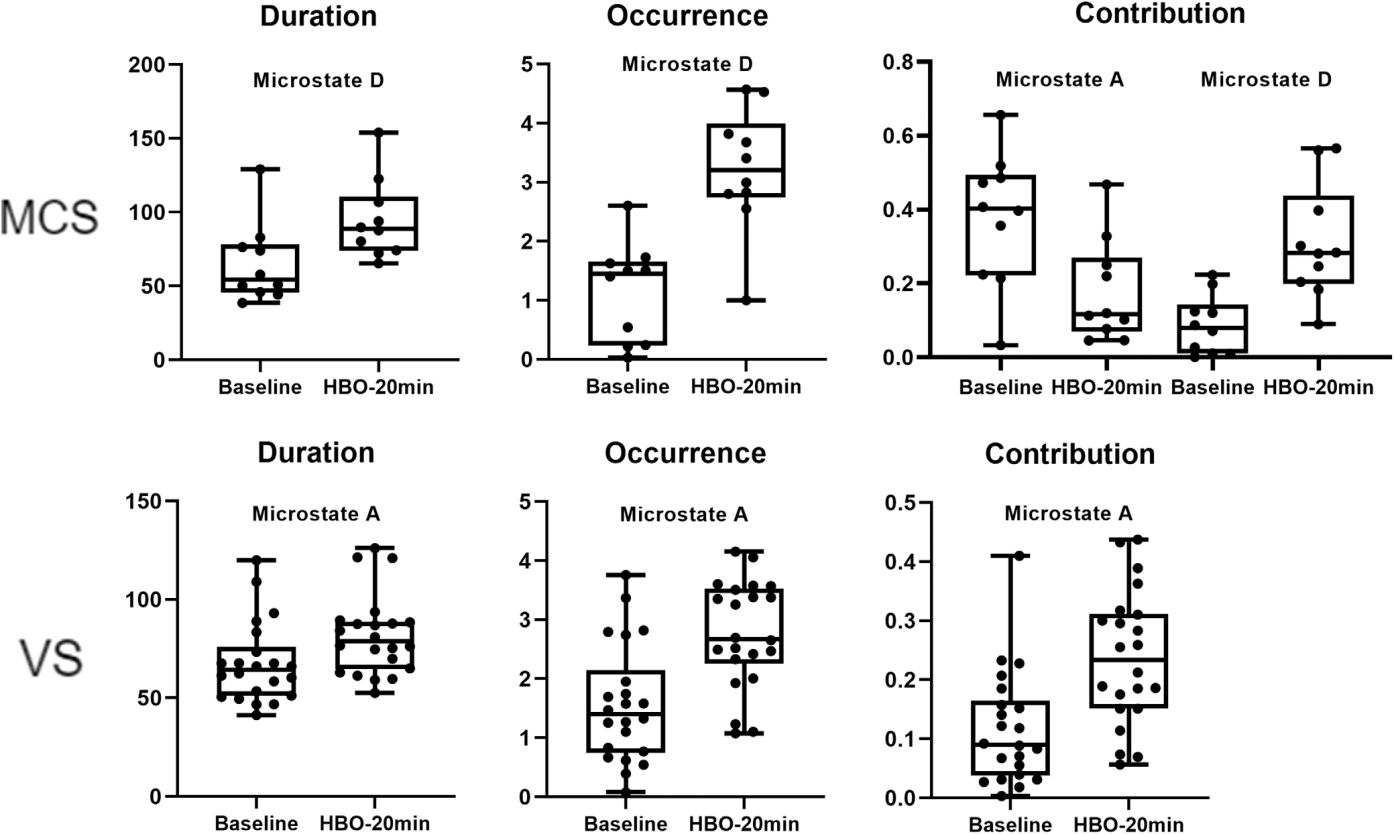
EEG microstate duration, occurrence, contribution, and mean GFP analysis results (*p* < 0.05) for patients in the MCS and UWS groups (32 patients).

**FIGURE 6:**
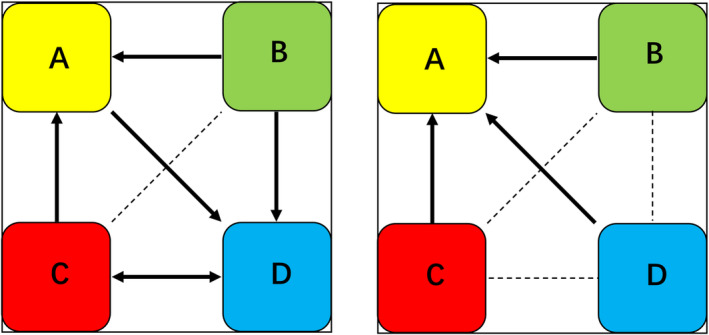
Schematic diagram of the mutual transition trends among the four microstates in the MCS (left) and UWS (right) groups. The four differently colored rectangles represent the four microstates. The dashed lines indicate nonsignificant transition trends between different microstates, and the solid arrows represent significant transition trends (*p* < 0.05).

Statistical analyses were performed on the EEG microstate parameters for both the MCS and UWS groups. Compared with pretreatment, after 20 min of HBO therapy, the MCS group presented a significant decrease in the coverage of microstate A, whereas there were significant increases in the duration, occurrence, and coverage of microstate D (Figures 3 and 5, Table 2). The transition probabilities from all microstates to microstate D, and from microstate D to C also significantly increased, whereas the transition probabilities from both microstate B and C to microstate A significantly decreased (Figure 6, Table 3). In the UWS group, patients showed a significant increase in the duration, occurrence, and coverage of microstate A after 20 min of treatment (Figures 4 and 5, Table 4). The transition probabilities from all microstates to microstate A are significantly increased (Figure 6, Table 5).

**TABLE 2:** EEG microstate duration, occurrence, contribution, and mean GFP analysis results of patients in the MCS group (10 patients).

	Class A		Class B		Class C		Class D	
	Baseline (mean ± SD)	HBO‐20 min (mean ± SD)	Baseline (mean ± SD)	HBO‐20 min (mean ± SD)	Baseline (mean ± SD)	HBO‐20 min (mean ± SD)	Baseline (mean ± SD)	HBO‐20 min (mean ± SD)
Duration (s)	0.104 ± 0.030	0.079 ± 0.039	0.137 ± 0.210	0.080 ± 0.033	0.104 ± 0.050	0.102 ± 0.022	0.065 ± 0.027	**0.095 ± 0.027**
Occurrence (s^−1^)	3.420 ± 3.078	2.099 ± 1.109	1.538 ± 0.850	1.950 ± 0.650	2.994 ± 1.210	3.378 ± 0.815	1.140 ± 0.836	**3.216 ± 1.048**
Contribution (%)	0.376 ± 0.179	**0.177 ± 0.138**	0.205 ± 0.270	0.165 ± 0.093	0.331 ± 0.208	0.347 ± 0.120	0.087 ± 0.079	**0.311 ± 0.155**
Mean GFP (mV)	6.258 ± 2.501	8.619 ± 7.886	6.571 ± 2.780	8.498 ± 7.491	6.382 ± 2.739	7.726 ± 4.527	7.005 ± 4.026	7.317 ± 3.975

*Note:* The bold values indicate that the *t* test result was significant (*p* < 0.05). Compared with the pretreatment values, the duration, occurrence and contribution of microstate D in the MCS group significantly increased after 20 min of HBO therapy (*p* < 0.05). Additionally, the contribution of microstate A was significantly reduced (*p* < 0.05). The other microstate parameters did not change significantly.

**TABLE 3:** EEG microstate transition probability analysis results for patients in the MCS group (10 patients).

	Class A		Class B		Class C		Class D	
	Baseline (mean ± SD)	HBO‐20 min (mean ± SD)	Baseline (mean ± SD)	HBO‐20 min (mean ± SD)	Baseline (mean ± SD)	HBO‐20 min (mean ± SD)	Baseline (mean ± SD)	HBO‐20 min (mean ± SD)
Class A (%)	—	—	0.479 ± 0.167	**0.237 ± 0.182**	0.617 ± 0.229	**0.255 ± 0.161**	0.426 ± 0.199	0.264 ± 0.158
Class B (%)	0.292 ± 0.270	0.0190 ± 0.112	—	—	0.260 ± 0.247	0.261 ± 0.147	0.242 ± 0.290	0.229 ± 0.081
Class C (%)	0.553 ± 0.264	0.415 ± 0.126	0.425 ± 0.195	0.392 ± 0.134	—	—	0.332 ± 0.176	**0.507 ± 0.145**
Class D (%)	0.155 ± 0.121	**0.395 ± 0.150**	0.096 ± 0.085	**0.371 ± 0.145**	0.122 ± 0.094	**0.484 ± 0.197**	—	—

*Note:* The bold values indicate that the *t* test result was significant (*p* < 0.05). The transition probabilities from microstate A to microstate C, microstate B to microstate D, microstate C to microstate D, and microstate D to microstate C were significantly greater after 20 min of HBO therapy than before HBO therapy (*p* < 0.05). The transition probability from microstate B to microstate A was significantly lower after 20 min of HBO therapy than before treatment (*p* < 0.05).

**TABLE 4:** EEG microstate duration, occurrence, contribution, and mean GFP analysis results of patients in the UWS group (22 patients).

	Class A		Class B		Class C		Class D	
	Baseline (mean ± SD)	HBO‐20 min (mean ± SD)	Baseline (mean ± SD)	HBO‐20 min (mean ± SD)	Baseline (mean ± SD)	HBO‐20 min (mean ± SD)	Baseline (mean ± SD)	HBO‐20 min (mean ± SD)
Duration (s)	0.067 ± 0.020	**0.082 ± 0.020**	0.089 ± 0.023	0.087 ± 0.024	0.102 ± 0.044	0.085 ± 0.017	0.092 ± 0.035	0.085 ± 0.020
Occurrence (s^−1^)	1.560 ± 0.993	**2.761 ± 0.907**	3.042 ± 0.985	2.803 ± 0.656	3.100 ± 0.768	2.906 ± 0.727	2.933 ± 0.899	2.925 ± 0.933
Contribution (%)	0.116 ± 0.095	**0.237 ± 0.113**	0.282 ± 0.124	0.249 ± 0.104	0.321 ± 0.165	0.253 ± 0.094	0.280 ± 0.147	0.261 ± 0120
Mean GFP (mV)	6.138 ± 2.543	6.295 ± 2.333	6.812 ± 2.922	6.229 ± 2.179	6.684 ± 2.966	6.315 ± 2.439	6.649 ± 2.506	6.546 ± 2.463

*Note:* The bold values indicate that the *t* test result was significant (*p* < 0.05). Compared with the pretreatment values, the duration, occurrence and contribution of microstate A in patients in the MCS group significantly increased after 20 min of HBO therapy (*p* < 0.05). The other microstate parameters did not change significantly.

**TABLE 5:** EEG microstate transition probability analysis results for patients in the UWS group (22 patients).

	Class A		Class B		Class C		Class D	
	Baseline (mean ± SD)	HBO‐20 min (mean ± SD)	Baseline (mean ± SD)	HBO‐20 min (mean ± SD)	Baseline (mean ± SD)	HBO‐20 min (mean ± SD)	Baseline (mean ± SD)	HBO‐20 min (mean ± SD)
Class A (%)	—	—	0.176 ± 0.132	**0.317 ± 0.142**	0.180 ± 0.134	**0.313 ± 0.133**	0.167 ± 0.113	**0.327 ± 0.114**
Class B (%)	0.319 ± 0.161	0.325 ± 0.129	—	—	0.411 ± 0.130	0.335 ± 0.143	0.399 ± 0.149	0.327 ± 0.111
Class C (%)	0.388 ± 0.177	0.330 ± 0.113	0.431 ± 0.147	0.350 ± 0.142	—	—	0.434 ± 0.182	0.347 ± 0.110
Class D (%)	0.293 ± 0.128	0.345 ± 0.115	0.393 ± 0.147	0.333 ± 0.149	0.409 ± 0.166	0.352 ± 0.146	—	—

*Note:* The bold values indicate that the *t* test result was significant (*p* < 0.05). The transition probabilities from microstate B, microstate C, and microstate D to microstate A were significantly greater after 20 min of HBO therapy than before treatment (*p* < 0.05).

### 3.4 HBO‐EEG Microstates in Patients With Different Prognoses

**FIGURE 7:**
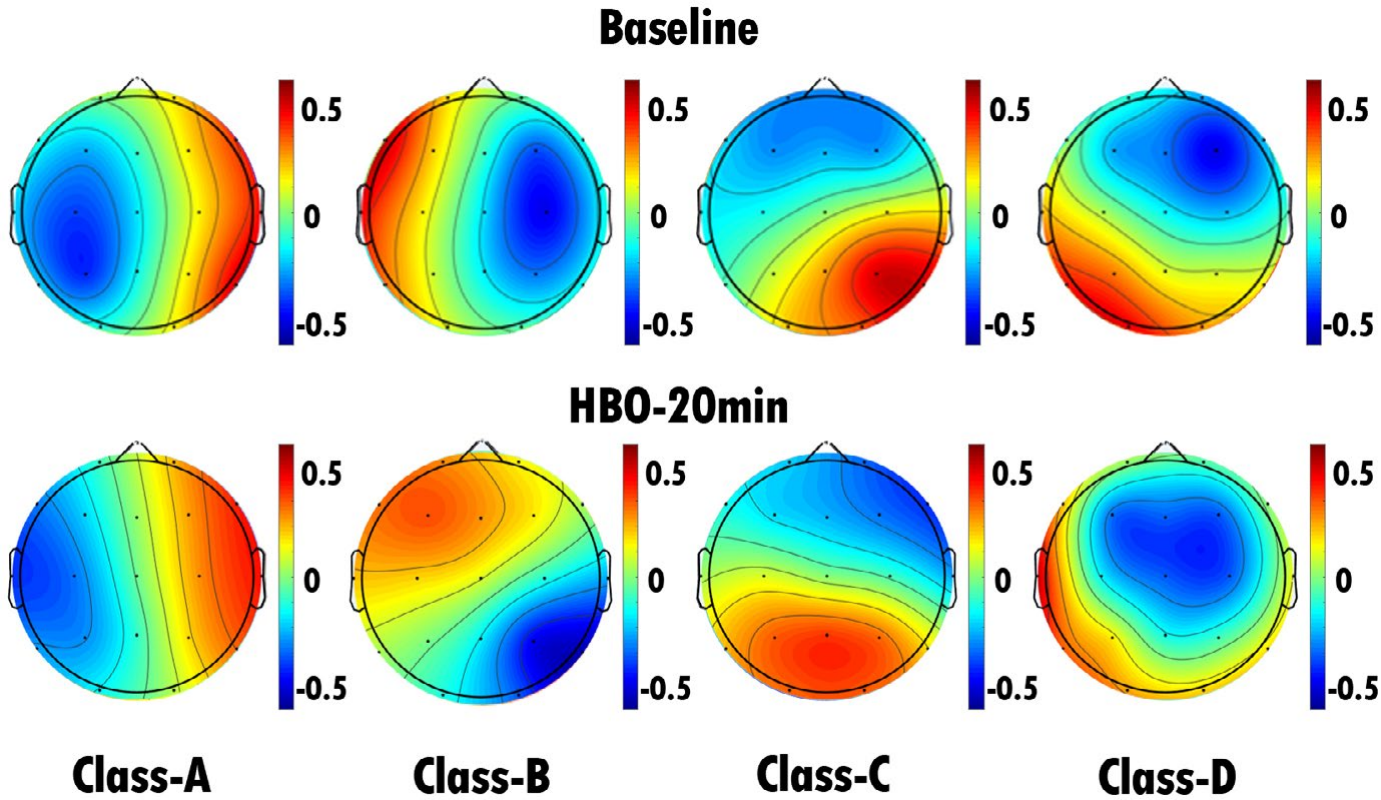
EEG microstate topography of patients in the improvement group (9 patients). The top row shows the EEG microstate topography map of the patients before HBO therapy, and the bottom row shows the EEG microstate topography map of the patients after 20 min of HBO therapy. Compared with the pretreatment values, there were significant increases in the duration, occurrence, and coverage of microstate D in the improvement group after HBO therapy.

**FIGURE 8:**
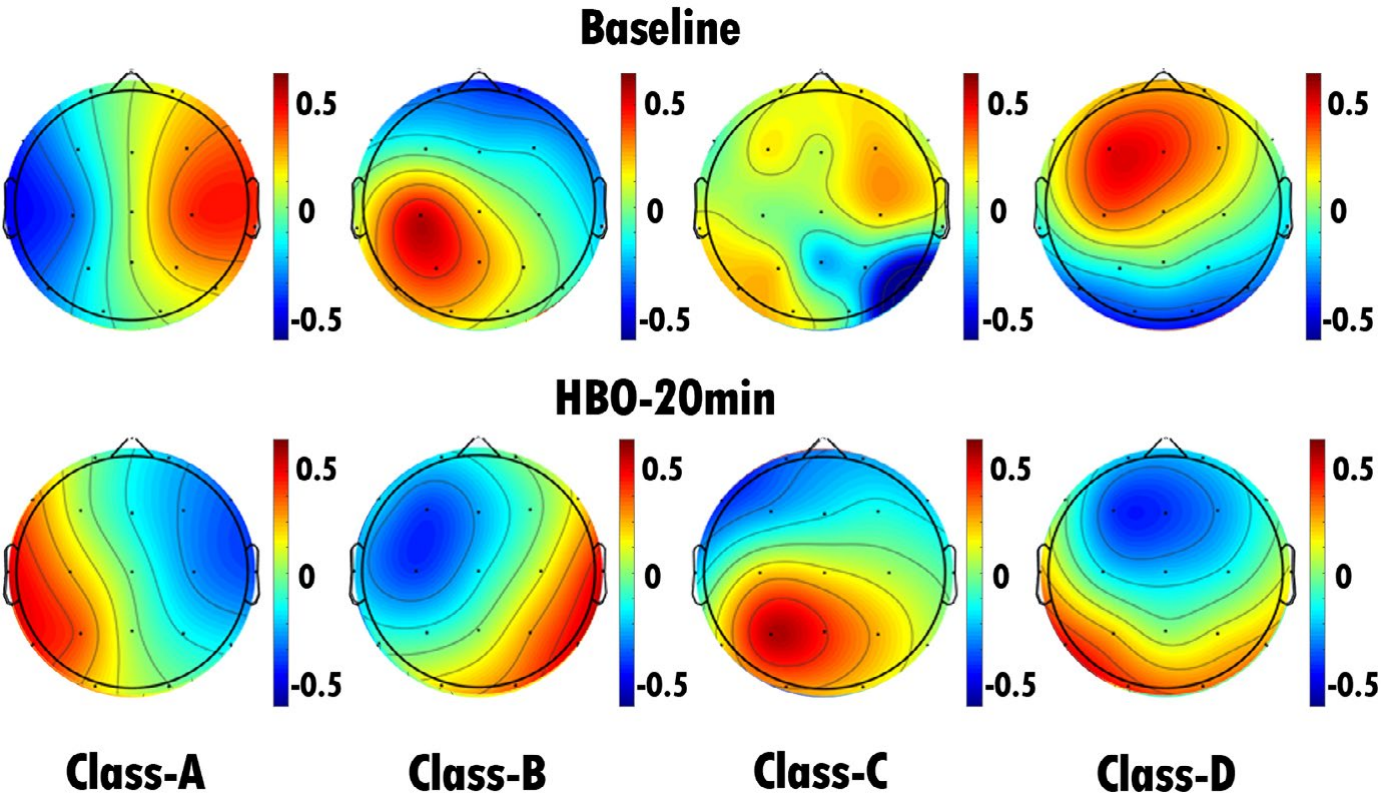
EEG microstate topography of patients in the nonimprovement group (23 patients). The top row shows the EEG microstate topography map of the patients before HBO therapy, and the bottom row shows the EEG microstate topography map of the patients after 20 min of HBO therapy. Compared with the pretreatment values, there were significant reductions in the duration and coverage of microstates A and C and significant increases in the occurrence and coverage of microstates B and D after 20 min of treatment.

**FIGURE 9:**
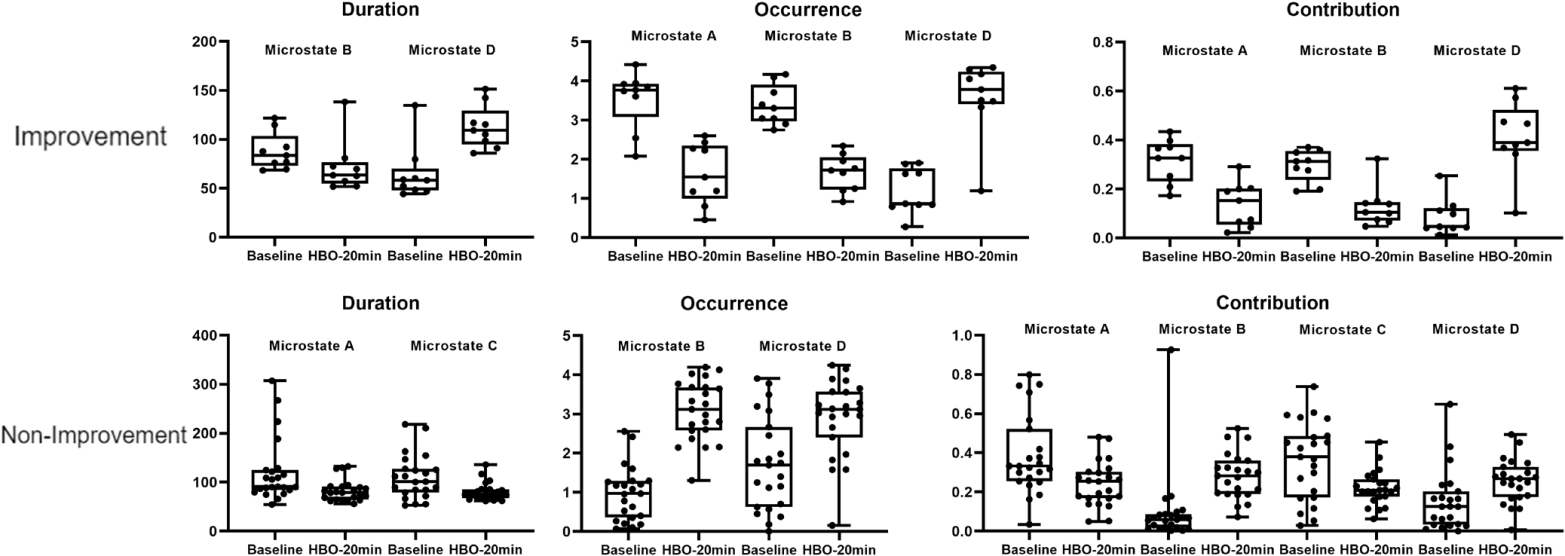
EEG microstate duration, occurrence, contribution, and mean GFP analysis results (*p* < 0.05) of the improvement and nonimprovement groups (32 patients).

**FIGURE 10:**
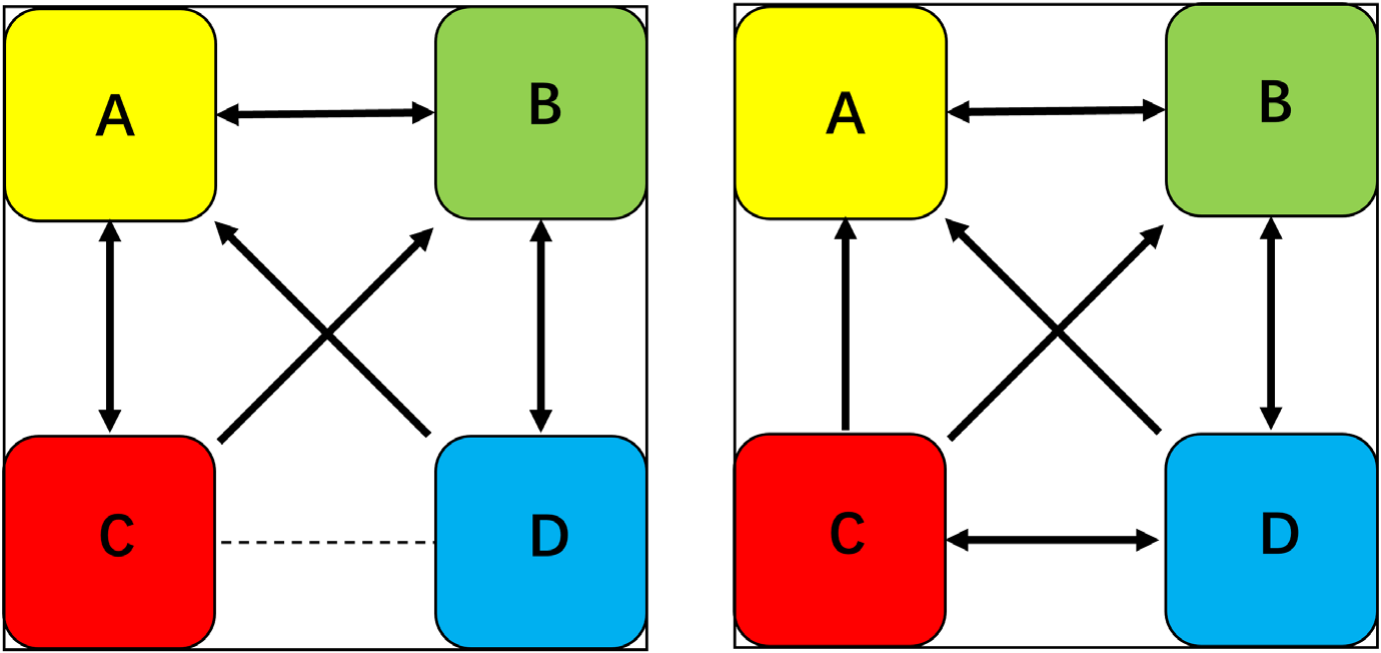
Schematic diagram of the mutual transition trends among the four microstates in the improvement group (left) and nonimprovement group (right). The four differently colored rectangles represent the four microstates. The dashed lines indicate nonsignificant transition trends between different microstates and the solid arrows represent significant transition trends (*p* < 0.05).

In our study, we conducted assessments of the severity of consciousness disorders in all included patients with TBI at admission and after 6 months of treatment. Relative to the CRS‐R scores at admission, patients who demonstrated an increase of ≥ 3 points in CRS‐R scores during follow‐up were categorized into the improvement group (nine patients), whereas those with an increase of less than three points or no improvement were categorized into the nonimprovement group (23 patients). We performed EEG microstate analysis on patients in both the improvement and nonimprovement groups before and during HBO therapy, resulting in the computation of microstate topographies at baseline and 20 min into HBO therapy (Figures 4 and 5). Statistical analyses were conducted on the EEG microstate parameters for the improvement and nonimprovement groups separately. The results revealed that for the improvement group, there were significant reductions in the occurrence and coverage of microstate A as well as the duration, occurrence, and coverage of microstate B after HBO therapy compared with the pretreatment values. Conversely, there were significant increases in the duration, occurrence, and coverage of microstate D in the improvement group after HBO therapy compared with the pretreatment values (Figures 7 and 9, Table 6). Furthermore, the transition probabilities between microstates A and B, from microstates C to A and B, from microstates D to A and B significantly decreased in the improvement group, whereas the transition probabilities from microstates B and C to D, and from D to C significantly increased (Table 7, Figure 10). In the nonimprovement group, there were significant reductions in the duration and coverage of microstates A and C and significant increases in the occurrence and coverage of microstates B and D after 20 min of treatment compared with the pretreatment values (Figures 8 and 9, Table 8). The transition probabilities from all microstates to microstate B, and microstate B to microstate D significantly increased, whereas the transition probabilities from all microstates to microstate A significantly decreased (Table 9, Figure 10).

**TABLE 6:** EEG microstate duration, occurrence, contribution, and mean GFP analysis results of improvement group patients (9 patients).

	Class A		Class B		Class C		Class D	
	Baseline (mean ± SD)	HBO‐20 min (mean ± SD)	Baseline (mean ± SD)	HBO‐20 min (mean ± SD)	Baseline (mean ± SD)	HBO‐20 min (mean ± SD)	Baseline (mean ± SD)	HBO‐20 min (mean ± SD)
Duration (s)	0.089 ± 0.013	0.078 ± 0.031	0.088 ± 0.019	**0.072 ± 0.027**	0.088 ± 0.010	0.098 ± 0.026	0.065 ± 0.028	**0.113 ± 0.022**
Occurrence (s^−1^)	3.540 ± 0.742	**1.633 ± 0.777**	3.378 ± 0.512	**1.662 ± 0.466**	3.426 ± 0.775	3.357 ± 0.808	1.187 ± 0.585	**3.573 ± 0.967**
Contribution (%)	0.317 ± 0.088	**0.138 ± 0.091**	0.296 ± 0.065	**0.128 ± 0.082**	0.301 ± 0.073	0.322 ± 0.069	0.087 ± 0.074	**0.412 ± 0.149**
Mean GFP (mV)	8.144 ± 2.671	9.411 ± 6.320	8.092 ± 1.926	10.371 ± 7.694	7.754 ± 2.025	10.024 ± 6.937	8.183 ± 3.353	8.658 ± 3.170

*Note:* The bold values indicate that the t test result was significant (*p* < 0.05). The duration, occurrence, and contribution of microstate B and the contribution and occurrence of microstate A were significantly lower after 20 min of HBO therapy than before HBO therapy (*p* < 0.05). The duration, occurrence, and contribution of microstate D significantly increased (*p* < 0.05). The other microstate parameters did not change significantly.

**TABLE 7:** EEG microstate transition probability analysis results for the improvement group (9 patients).

	Class A		Class B		Class C		Class D	
	Baseline (mean ± SD)	HBO‐20 min (mean ± SD)	Baseline (mean ± SD)	HBO‐20 min (mean ± SD)	Baseline (mean ± SD)	HBO‐20 min (mean ± SD)	Baseline (mean ± SD)	HBO‐20 min (mean ± SD)
Class A (%)	—	—	0.444 ± 0.091	**0.187 ± 0.111**	0.437 ± 0.066	**0.175 ± 0.093**	0.460 ± 0.110	**0.213 ± 0.115**
Class B (%)	0.376 ± 0.101	**0.209 ± 0.144**	—	—	0.441 ± 0.081	**0.185 ± 0.119**	0.425 ± 0.088	**0.216 ± 0.066**
Class C (%)	0.305 ± 0.090	0.351 ± 0.096	0.427 ± 0.100	0.396 ± 0.092	—	—	0.115 ± 0.076	**0.571 ± 0.149**
Class D (%)	0.320 ± 0.102	0.439 ± 0.125	0.129 ± 0.082	**0.416 ± 0.128**	0.122 ± 0.071	**0.640 ± 0.192**	—	—

*Note:* The bold values indicate that the *t* test result was significant (*p* < 0.05).

**TABLE 8:** EEG microstate duration, occurrence, contribution, and mean GFP analysis results of patients in the nonimprovement group (23 patients).

	Class A		Class B		Class C		Class D	
	Baseline (mean ± SD)	HBO‐20 min (mean ± SD)	Baseline (mean ± SD)	HBO‐20 min (mean ± SD)	Baseline (mean ± SD)	HBO‐20 min (mean ± SD)	Baseline (mean ± SD)	HBO‐20 min (mean ± SD)
Duration (s)	0.121 ± 0.065	**0.083 ± 0.022**	0.091 ± 0.152	0.089 ± 0.022	0.111 ± 0.045	**0.081 ± 0.018**	0.075 ± 0.030	0.085 ± 0.017
Occurrence (s^−1^)	3.199 ± 0.818	2.789 ± 0.849	0.967 ± 0.686	**3.084 ± 0.765**	3.064 ± 1.105	2.671 ± 0.671	1.716 ± 1.187	**2.911 ± 0.965**
Contribution (%)	0.387 ± 0.202	**0.241 ± 0.115**	0.098 ± 0.187	**0.283 ± 0.117**	0.362 ± 0.197	**0.221 ± 0.088**	0.153 ± 0.155	**0.256 ± 0.112**
Mean GFP (mV)	6.101 ± 2.919	5.785 ± 2.319	5.455 ± 2.319	5.798 ± 2.332	6.247 ± 2.848	5.717 ± 2.360	5.820 ± 2.510	6.103 ± 2.492

*Note:* The bold values indicate that the *t* test result was significant (*p* < 0.05). The duration and contribution of microstates A and C were significantly lower after 20 min of HBO therapy than before HBO therapy (*p* < 0.05). The occurrence and contribution of microstates B and D significantly increased (*p* < 0.05). The other microstate parameters did not change significantly.

**TABLE 9:** EEG microstate transition probability analysis results for patients in the nonimprovement group (23 patients).

	Class A		Class B		Class C		Class D	
	Baseline (mean ± SD)	HBO‐20 min (mean ± SD)	Baseline (mean ± SD)	HBO‐20 min (mean ± SD)	Baseline (mean ± SD)	HBO‐20 min (mean ± SD)	Baseline (mean ± SD)	HBO‐20 min (mean ± SD)
Class A (%)	—	—	0.445 ± 0.202	**0.329 ± 0.130**	0.616 ± 0.204	**0.312 ± 0.145**	0.489 ± 0.214	**0.341 ± 0.120**
Class B (%)	0.171 ± 0.211	**0.369 ± 0.126**	—	—	0.141 ± 0.168	**0.377 ± 0.144**	0.111 ± 0.165	**0.366 ± 0.123**
Class C (%)	0.0580 ± 0.226	**0.295 ± 0.124**	0.386 ± 0.184	0.317 ± 0.117	—	—	0.340 ± 0.236	0.293 ± 0.093
Class D (%)	0.250 ± 0.190	0.336 ± 0.109	0.169 ± 0.139	**0.354 ± 0.135**	0.243 ± 0.201	0.311 ± 0.132	—	—

*Note:* The bold values indicate that the *t* test result was significant (*p* < 0.05).

We aimed to conduct a more in‐depth analysis of TBI patients by analyzing the EEG microstate parameters of both the improvement group and the MCS group. The results indicated that there was a significant increase in the duration of microstate D, as well as in the transition probabilities from microstate C to microstate D and from microstate D to microstate C after 20 min of HBO therapy in both the improvement group and the MCS group.

## 4 Discussion

To date, various behavioral, neuroimaging, and electrophysiological methods have been employed to assess the level of consciousness in patients with pDOC [29]. EEG is an important electrophysiological technique that is widely applied to evaluate brain function in patients with pDOC [30]. Microstate analysis is used to examine scalp electroencephalographic topological structures from a global pattern perspective and can reveal systematic temporal dynamic changes. Microstate analysis can also detect different states of the brain, thus providing a unique perspective for understanding patients with pDOC [31, 32].

We monitored EEG signals during HBO therapy in 32 patients with TBI included in the present study. Conducting real‐time EEG monitoring studies under HBO therapy is both safe and feasible. This approach not only offers an objective basis for precise HBO therapy but also enhances the clinical assessment indicators for pDOC. By performing microstate analysis of EEG signals before and during HBO therapy, this study revealed significant differences in certain microstate indicators among TBI patients.

In previous research, we studied 41 patients with varying degrees of DOC during HBO therapy. The results indicated that after 20 min of HBO therapy, the duration of microstate C increased in all DOC patients and in the high CRS‐R group. The transition probabilities between microstate A and microstate C also significantly increased in the high‐CRS‐R group during HBO therapy [12].

In contrast to previous studies with a broader range of aetiologies [33, 34, 35], our current study specifically included 32 pDOC patients with TBI as the cause. We collected baseline information and conducted real‐time EEG monitoring during HBO therapy. Our research demonstrated a greater degree of specificity, a broader spectrum of consciousness levels, and more representative results than similar studies on DOC [36, 37, 38].

### 4.1 Microstate A and D Patterns in pDOC (UWS and MCS)

The clinical distinction between the MCS and UWS relies primarily on the CRS‐R score, with the misdiagnosis rate reaching as high as 40% [26]. We employed EEG analysis, which serves as a pivotal auxiliary tool for diagnosing patients with DOC. At that time, a conclusive decision regarding the clinical diagnosis of DOC patients in the realm of microstates had not been reached. Thus, microstates serve as a potential complement to vital electrophysiological indicators for assessing consciousness.

The parameters of microstate A in the UWS group showed notable improvement, accompanied by a pronounced increase in transitions from microstates B, C, and D to microstate A. Microstate A is primarily concentrated in the bilateral temporal regions, including the superior and middle temporal gyri, and is involved in the initial processing of auditory information [27]. Certain brain functions are intricately linked to language and auditory processing capabilities. This finding indicated that, during HBO therapy, patients in the UWS group were more engaged in the input and processing of auditory information than were those in the untreated state, thus indicating a degree of activation within the auditory network. Furthermore, on the basis of the microstate parameters presented in the table, compared with those of other patients, the microstate A parameters of patients in the UWS group were considerably lower before HBO therapy.

In contrast, patients in the MCS group exhibited a marked decline in all parameters associated with microstate A, including a significant reduction in transition probabilities from other microstates to microstate A. Concurrently, the parameters of microstate D showed a substantial increase, coupled with a noticeable increase in transition probabilities from other microstates to microstate D. Microstate D, which is known to be linked with the central executive network and responsible for functions such as task selection and decision‐making in higher‐order cognitive tasks [21], presented increased activity during microstate analysis. Specifically, regions within the right frontal and parietal lobes, both dorsal and ventral, associated with microstate D in patients in the MCS group demonstrated heightened activity throughout HBO therapy. Consequently, we inferred that during this therapy, patients with diminished consciousness levels experienced these changes.

In our previous study [12], we included 43 patients with DOC, among whom 19 patients with CRS‐R scores ≥ 8 presented a significant increase in the duration of microstate C and the transition probabilities between microstates A and C after 20 min of HBO therapy. However, in the present study, we found different patterns: patients in the MCS group presented significant increases in the parameters of microstate D during HBO therapy, whereas patients in the UWS group presented significant increases in the parameters of microstate A. This difference may be attributed to the fact that our current study focused primarily on pDOC patients with TBI, whereas previous research included pDOC patients with more complex aetiologies. Nevertheless, when the results of both studies were combined, we observed that patients with higher levels of consciousness demonstrated significant increases in the parameters of microstates C and D following HBO therapy. Since microstates C and D are associated with more advanced and complex brain networks, these findings also support the notion that patients with better consciousness levels exhibit increased activation of cortical regions related to increased cognitive functions during recovery [39].

### 4.2 Changes in the Parameters of Microstate D With Different Recoveries in pDOC

Although patients with pDOC are divided into MCS and UWS groups, their clinical outcomes are more valuable for research.

In the improvement group, patients displayed a significant decrease in various parameters of microstates A and B. This resulted in a notable reduction in the transition probabilities from other microstates to microstates A and B. Additionally, there were significant increases in the parameters of microstate D, along with a marked increase in the transition probabilities from other microstates to microstate D.

This finding is similar to the recent findings of Guo et al. [34], who noted that in patients in both the UWS and MCS groups who were receiving HD‐tDCS treatment, the occurrence probability per second (OPS) of microstate D was positively correlated with CRS‐R scores. As the OPS of microstate D increases, patients transition from the UWS to the MCS. This finding suggests a potential association between microstate D and recovery in patients with a higher level of consciousness [40]. This finding is consistent with the EEG microstate findings observed in our clinical observations.

The overall trends in the changes in microstate parameters in both patient groups were similar, which may have been related to the severity of the patient's conditions and levels of consciousness or potentially associated with an insufficient duration of HBO.

### 4.3 Microstate D Patterns in “Good” Patients With pDOC

In the clinical management of patients diagnosed with pDOC, the prevailing belief is that those clinically diagnosed with MCS have a more favorable prognosis and recovery. Through an examination of EEG changes during HBO therapy, common features consistent with clinical observations were identified. The results substantiated the prevailing consensus that there were shared alterations in the microstates of patients in the MCS group, including within both the treatment and improvement subgroups. This finding serves as compelling evidence supporting the subsequent recovery of patients diagnosed with MCS.

Compared with pre‐HBO therapy, after 20 min of HBO therapy, patients in the improvement group presented a significant increase in the duration of microstate D, as well as a notable increase in the transition probabilities between microstates C and D. Similar trends were observed in patients in the MCS group. Since microstate D is believed to be associated with the central executive network in the brain, the significant increases in the parameters of microstate D under HBO conditions suggested a certain level of activation in brain regions responsible for increased cognitive functions such as attention and task decision‐making. Moreover, the increased transition probabilities between microstates C and D indicated enhanced activity in cognitive functions and sensory information processing in patients compared with before treatment. Therefore, on the basis of these findings, patients with a greater level of consciousness were more likely to have a better prognosis with HBO therapy.

Our findings are consistent with the findings of Toplutaş, Aydın, and Hanoğlu [35], who conducted a comparative study involving 28 DOC patients and 18 healthy controls. They reported that the parameter characteristics of microstate D were closely associated with clinical scale scores, thereby demonstrating that microstate D is the most accurate parameter for representing consciousness level. This finding suggests a strong connection between microstate D and the level of consciousness as well as clinical recovery in patients with DOC [41, 42].

## 5 Limitations

This study has several limitations. First, the potential influence of various therapies received by patients, considering their complex complications, might not have been fully considered. Additionally, we did not account for the potential impact of varying diets. Second, the sample size of our study is relatively small, and despite the sample size limitation, the consistency of our results is strong. The predictive accuracy of the biomarkers we identified requires external validation and verification through other methods. Finally, investigating the source location of EEG microstates may require a higher spatial resolution. In addition to the four classical microstates described above, some other classes also need to be considered, as they might influence brain functions.

## 6 Conclusion

Real‐time EEG detection in patients with pDOC during HBO therapy is safe and feasible. This study utilized EEG microstate analysis during HBO therapy in patients with TBI and demonstrated regional activation in specific brain areas in response to HBO therapy, which plays a role in the recovery of consciousness levels in patients. We found that consciousness level was correlated with microstates A and D, with a more substantial change observed in microstate D among patients with better clinical outcomes. Additionally, both patient groups exhibited a common characteristic: significant increases in the parameters of microstate D after HBO therapy. Microstate D may be associated with the recovery of consciousness levels in patients. EEG changes during HBO therapy serve as a significant complement to existing consciousness grading methods and prognostic evaluations.

## Author Contributions

Long Xu: conceptualization, data curation, methodology, investigation, writing – original draft, funding acquisition. Jiameng Wang: conceptualization, data analysis, formal analysis, writing – original draft. Cong Wang: data collection. Qianqian Ge: writing – review and editing. Ziqi Ren: project administration, investigation. Chen He: writing – review and editing. Yun Liu: data analysis, formal analysis. Bo Wang: supervision. Yaling Liu: data collection. Lianbi Xue: data collection. Jianghong He: supervision, funding acquisition. Xudong Zhao: conceptualization, supervision, software, funding acquisition. Qiuhong Yu: conceptualization, supervision, resources, funding acquisition. We confirm that the manuscript has been read and approved by all named authors and that there are no other persons who satisfied the criteria for authorship but are not listed. We further confirm that the order of the authors listed in the manuscript has been approved by all of us.

## Ethics Statement

We further confirm that any aspect of the work covered in this manuscript involving human patients has been conducted with the ethical approval of all relevant bodies and that such approvals are acknowledged within the manuscript.

## Conflicts of Interest

The authors declare no conflicts of interest.

## Data Availability

The data that support the findings of this study are available upon request from the corresponding author. The data are not publicly available due to privacy or ethical restrictions.
